# Association of *CCND1* rs9344 polymorphism with lung cancer susceptibility and clinical outcomes: a case-control study

**DOI:** 10.1186/s12890-024-02983-1

**Published:** 2024-04-08

**Authors:** Chao Mei, Tian Wang, Baoli Xu, Sanlan Wu, Xuelin Zhang, Yongning Lv, Yu Zhang, Zhaoqian Liu, Weijing Gong

**Affiliations:** 1 Department of Pharmacy, Union Hospital, Tongji Medical College, Huazhong University of Science and Technology, Wuhan, China; 2 Department of General medicine, Huangshi Central Hospital, The Affifiliated Hospital of Hubei Polytechnic University, Huangshi, China; 3 People’s Hospital Of Chong Qing Liang Jiang New Area, Chongqing, China; 4 Department of Clinical Pharmacology, Hunan Key Laboratory of Pharmacogenetics, National Clinical Research Center for Geriatric Disorders, Xiangya Hospital, Central South University, Changsha, China; 5 Hubei Province Clinical Research Center for Precision Medicine for Critical Illness, Wuhan, China

**Keywords:** Lung cancer, *CCND1* rs9344, Platinum-based chemotherapy, Susceptibility, Prognosis

## Abstract

**Background:**

Cyclin D1 (*CCND1*) plays a pivotal role in cancer susceptibility and the platinum-based chemotherapy response. This study aims to assess the relationship between a common polymorphism (rs9344 G > A) in *CCND1* gene with cancer susceptibility, platinum-based chemotherapy response, toxicities and prognosis of patients with lung cancer.

**Methods:**

This study involved 498 lung cancer patients and 213 healthy controls. Among them, 467 patients received at least two cycles of platinum-based chemotherapy. Unconditional logistical regression analysis and meta-analysis were performed to evaluate the associations.

**Results:**

The lung adenocarcinoma risk was significantly higher in patients with AA than GG + GA genotype (adjusted OR = 1.755, 95%CI = 1.057–2.912, *P* = 0.030). *CCND1* rs9344 was significantly correlated with platinum-based therapy response in patients receiving PP regimen (additive model: adjusted OR = 1.926, 95%CI = 1.029–3.605, *P* = 0.040; recessive model: adjusted OR = 11.340, 95%CI = 1.428–90.100, *P* = 0.022) and in the ADC subgroups (recessive model: adjusted OR = 3.345, 95%CI = 1.276–8.765, *P* = 0.014). Furthermore, an increased risk of overall toxicity was found in NSCLC patients (additive model: adjusted OR = 1.395, 95%CI = 1.025–1.897, *P* = 0.034; recessive model: adjusted OR = 1.852, 95%CI = 1.088–3.152, *P* = 0.023), especially ADC subgroups (additive model: adjusted OR = 1.547, 95%CI = 1.015–2.359, *P* = 0.043; recessive model: adjusted OR = 2.030, 95%CI = 1.017–4.052, *P* = 0.045). Additionally, *CCND1* rs9344 was associated with an increased risk of gastrointestinal toxicity in non-smokers (recessive model: adjusted OR = 2.620, 95%CI = 1.083–6.336, *P* = 0.035). Non-significant differences were observed in the 5-year overall survival rate between *CCND1* rs9344 genotypes. A meta-analysis of 5432 cases and 6452 control samples did not find a significant association between lung cancer risk and *CCND1* rs9344 polymorphism.

**Conclusion:**

This study suggests that in the Chinese population, CCND1 rs9344 could potentially serve as a candidate biomarker for cancer susceptibility and treatment outcomes in specific subgroups of patients.

**Supplementary Information:**

The online version contains supplementary material available at 10.1186/s12890-024-02983-1.

## Background

Lung cancer is a prevalent disease that seriously endangers global public health [1–4]. According to statistics, there were about 2.20 million newly-diagnosed lung cancer cases and 1.79 million mortalities worldwide every year [4, 5]. Lung cancer accounts for more than 20% of cancer-related deaths worldwide, surpassing the combined mortality rates of prostate, breast, and colon cancers [1, 6–8]. Despite the progress made in targeted therapy and immunotherapy in the recent decades, platinum-based chemotherapy remains the most widely used treatment option in clinical practice [9–12]. However, due to individual variations in sensitivity, only a subset of patients benefits from this treatment [13]. Given the potential toxic reactions, it is urgent to discover reliable predictive biomarkers to predict the prognosis, therapeutic efficacy and toxicity of lung cancer patients, which is crucial for promoting personalized medicine and enhancing therapeutic outcomes [14–16].

Cyclins D1 (*CCND1*) plays a vital role in cell cycle regulation which mediates the G1 to S phase transition [17–19]. It also has a fundamental involvement in human cancer progression, including cell proliferation, transcription, chromosome duplication and stability, DNA damage response, metabolism, tumor migration and invasion [17, 20, 21]. Multiple clinical studies demonstrated that dysregulation of *CCND1* is associated with poor prognosis and platinum-based chemotherapy response in various human cancers, highlighting its potential as a tumor predictive biomarker [22–32].

Single nucleotide polymorphisms (SNPs) refer to DNA sequence polymorphisms caused by single nucleotide variation at the genomic level, accounting for over 90% of all known polymorphisms [33–35]. Cyclins D1 is the second most frequently amplified locus in human solid tumors [36, 37]. The association between *CCND1* A870G (rs9344) polymorphism and cancer risk has been previously investigated in lung cancer [38–43]. However, due to the limited number of studies and sample size, the exact role of *CCND1* polymorphism in predicting lung cancer risk remains unclear. Only few studies have been conducted to investigate the correlation between *CCND1* rs9344 and platinum-based chemotherapy response in lung cancer.

This study aimed to investigate the association of *CCND1* rs9344 with cancer susceptibility, platinum-based chemotherapy, toxicity and overall survival of patients with lung cancer by performing hospital-based case-control study. Additionally, a meta-analysis was conducted using 5432 cases and 6452 control samples to evaluate the association between *CCND1* rs9344 polymorphism and lung cancer risk. The results may provide evidence in support of the potential utilization of *CCND1* rs9344 as a predictive biomarker for prognosis and chemotherapy sensitivity in Chinese patients with lung cancer in certain conditions.

## Methods

### Study design

#### Setting

During November 2011 to May 2013, 498 patients with primary lung cancer (diagnosed by cytology or histology) were consecutively recruited at Xiangya Hospital and the Affiliated Cancer Hospital of Central South University in Changsha, Hunan Province, China. During the same period, 213 healthy controls were collected from the physical examination center of Xiangya Hospital of Central South University. This study was approved by the Ethics Committee of Xiangya School of Medicine, Central South University (registration number: CTXY-110008-2), and all subjects enrolled have signed the informed consent.

### Participants

All patients had been histologically or cytologically confirmed to have primary lung cancer. Subjects who were pregnant, lactating, had active infections, symptomatic brain or leptomeningeal metastases, or other previous or concurrent malignancies were excluded from the study. Among them, 467 patients were enrolled in the platinum-based chemotherapy response study. The inclusion criteria were as follows: (1) They were not administered radiotherapy and/or biological therapy prior to or during chemotherapy; (2) they received at least two cycles of platinum-based chemotherapy; (3) they underwent full follow-up (to March 2017); (4) tumors were assessed before and during treatment using the same imaging methods (Supplementary Table 1). Platinum-based chemotherapy regimens include pemetrexed + platinum (PP), gemcitabine + platinum (GP), paclitaxel + platinum (TP), docetaxel + platinum (DP), etoposide + platinum (EP), and other platinum-based chemotherapy regimens (irinotecan + platinum, navibine + platinum). In the case of the healthy controls, individuals with a smoking history, a history of lung ailments, or those engaged in high-risk occupations such as chemical, construction, asbestos, and coal mining work were excluded.

### Variables

The endpoints of the study were as follows: chemotherapy response was evaluated based on the Response Evaluation Criteria in Solid Tumors (RECIST) guidelines and categorized as responders (complete response: CR, partial response: PR) or non-responders (stable disease: SD and progressive disease: PD). Two professional radiologists independently evaluated the CT scans of lung cancer patients before and after chemotherapy to assess the treatment effectiveness after two cycles of therapy. In case of disagreement, a third radiologist was consulted. Toxicity was assessed according to the National Cancer Institute Common Toxicity Criteria 3.0 during the first two cycles of chemotherapy regimen. Grade 3 or 4 toxicity was defined as severe toxicity. Severe gastrointestinal toxicity was grade 3 or 4 nausea and vomiting. Severe hematological toxicity included grade 3 or 4 hypochromia, leukopenia, neutropenia and thrombocytopenia. Patients who experienced any type of the grade 3 or 4 toxicities described above were defined as suffering severe overall toxicity.

For the lung caner patients, age, sex, smoking status, stage, histological type, and chemotherapy regimens were collected. For the healthy controls, age, sex and smoking status were collected. The above factors age, sex, smoking status, stage, histological type, and chemotherapy regimens were considered as covaraites in this study.

### DNA extraction and genotyping analysis

Venous blood DNA was extracted using the Genomic DNA Purification Kit (Promega, Madison, WI, USA). *CCND1* rs9344 was genotyped using the Sequenom MassARRAY System (Sequenom, San Diego, CA, USA).

### Study selection and data extraction criteria of meta-analysis

The Pubmed, Embase and Cochrane databases were utilized to identify original studies examing the association between *CCND1* rs9344 and lung cancer susceptibility (up to March 29, 2023). The search formula was: “CCND1 or Cyclin D1” and “genetic polymorphism or polymorphisms or variant or rs9344” and “lung cancer”. Included studies had to be original case-control studies with detailed *CCND1* rs9344 genotype frequencies or available data. The qualities of selected studies were independently assessed and identified by two researchers. The following information was extracted from the included studies: the last name of the first author, year of publication, country, ethnicity, cancer type, source of cases and controls, number of cases and controls, genotyping method, genotype or allele frequency, and HWE *p* values for controls.

### Statistical analysis

The study size was estimated using Power Analysis and Sample Size (PASS) 2021 (NCSS, LLC. Kaysville, Utah, USA) at a power value of 0.80. The chi-square test was used to assess differences in proportions between groups for the categorical variables. The median age of lung cancer patients, 57 years old, was used as cut-off value. The Hardy-Weinberg equilibrium was calculated using the chi-square test. Associations between *CCND1* rs9344 and cancer susceptibility, therapeutic response and toxicity were estimated by unconditional logistic regression. Factors including age, sex, smoking status, stage, histological type, and chemotherapy regimens were considered as covaraites in this study. Survival curves were calculated using the Kaplan-Meier method, and survival analyses were conducted using Cox proportional hazards regression analysis. All significance tests were two-sided, and *P <* 0.05 was defined as statistically significant. The above analyses were performed using PLINK 1.9 and PASW statistics v18.0 (IBM Co., Armonk, NY, USA).

In the meta-analysis, the association between cancer risk and *CCND1* rs9344 was assessed by calculating pooled OR and 95% CI. The heterogeneity of the effect size across studies was estimated and quantified by Cochrane’s *Q* test and *I*^*2*^ test. The random effect model is selected if *P <* 0.1 or *I*^*2*^ > 50%, otherwise, the fixed effect model is adopted. The stability of the results was assessed by sensitivity analysis. The inverted funnel plot was used to estimate the publication bias. All statistical analysis was performed in R4.2.3. *P* < 0.05 was considered statistically significant.

## Results

###  Participants and descriptive data

In this study, 498 cases of lung cancer (394 males and 104 females) and 213 healthy controls (80 males and 133 females) were included. The clinical characteristics of the participants, including sex, age, histology, tumor stage, regimen, therapeutic response and toxicities were listed in Table 1 and Supplementary Table 1. The genotype distribution of *CCND1* rs9344 was in agreement with the Hardy Weinberg equilibrium (*P* = 0.539).

**Table 1:** Demographics of lung cancer patients and healthy controls

Characteristics	Patients, n(%)	Controls, n(%)	*P*
	(*n* = 498)	(*n* = 213)	
**Sex**			
Male	394(79.1)	80(37.6)	0.000^*^
Female	104(20.9)	133(62.4)	
**Age (years)**			
< 57	242(48.6)	74(34.7)	0.000^*^
≥ 57	256(51.4)	139(65.3	
**Histology**			
NSCLC	429(86.1)		
SCLC	69(13.9)		
SCC	189(37.9)		
ADC	217(43.6)		
Other^a^	23(4.6)		
**Stage (NSCLC)**			
I, II	13(3.0)		
III, IV	416(97.0)		
**Stage (SCLC)**			
Limited	36(52.2)		
Extensive	33(47.8)		
**Regimen**			
Regimen1	192(41.4)		
Regimen2	68(14.6)		
Regimen3	137(29.3)		
Regimen4	27(5.8)		
Regimen5	29(6.2)		
Other^b^	14(3.0)		
**Chemotherapy response**	467		
Responder	283(60.6)		
Non-responder	184(39.4)		
**Overall toxicity**	467		
Grade 0–2	286(61.2)		
Grade 3–4	181(38.8)		
**Gastrointestinal toxicity**	467		
Grade 0–2	366(78.4)		
Grade 3–4	101(21.6)		
**Hematological toxicity**	467		
Grade 0–2	353(75.6)		
Grade 3–4	114(24.4)		

*Abbreviations* *n *number, *SCC* Squamous cell carcinoma, *ADC *Adenocarcinoma, *SCLC *Small cell lung cancer

Other^a^ mixed-cell or undifferentiated carcinoma, NSCLC Non-small cell lung cancer, Regimen1 platinum + gemcitabine,  Regimen2 Platinum + etoposide, Regimen3 Platinum + pemetrexed, Regimen4 Platinum + paclitaxel, Regimen5 Platinum + docetaxel,

Other^b^ platinum + irinotecan or platinum + navelbine

^*^P < 0.05

### Association between *CCND1* rs9344 and lung cancer susceptibility

After adjusting for age and sex, the association between *CCND1* rs9344 polymorphism and cancer risk was analyzed in additive, dominant and recessive models, respectively. The results of logistic regression analysis were shown in Table 2 and Supplementary Tables 2, and the OR values with 95%CI in different genetic models were as follows: additive model (GG vs. GA vs. AA: adjusted OR = 1.115, 95%CI = 0.869–1.431, *P* = 0.391); dominant model (GA + AA vs. GG: adjusted OR = 0.980, 95%CI = 0.673–1.425, *P* = 0.914); recessive model (AA vs. GG + GA: adjusted OR = 1.498, 95%CI = 0.935–2.399, *P* = 0.0927). These results did not indicate a significant correlation between *CCND1* rs9344 and the risk of lung cancer.

**Table 2:** Association of *CCND1* rs9344 with cancer susceptibility and clinical outcomes in patients received platinum-based chemotherapy

Type	Genotype	n (%)	n (%)	Additive model		Dominant model		Recessive model	
				OR (95% CI)	*P*	OR (95% CI)	*P*	OR (95% CI)	*P*
Susceptiblity^a^		Case	Control	1.115(0.869–1.431)	0.391	0.980(0.673–1.425)	0.914	1.498(0.935–2.399)	0.0927
	GG	127(25.5)	33(15.5)						
	GA	237(47.6)	106(49.8)						
	AA	126(25.3)	72(33.8)						
Chemotherapy response^b^		Responder	Non-responder	1.225(0.934–1.607)	0.142	1.274(0.848–1.914)	0.243	1.375(0.838–2.255)	0.207
	GG	31(16.8)	61(21.6)						
	GA	85(46.2)	134(47.3)						
	AA	67(36.4)	85(30.0)						
Overall toxicity^b^		Grade 0–2	Grade 3–4	1.142(0.874–1.493)	0.33	1.110(0.736–1.674)	0.618	1.323(0.824–2.125)	0.246
	GG	51(17.8)	41(22.7)						
	GA	137(47.9)	83(45.9)						
	AA	94(32.9)	57(31.5)						
Gastrointestinal toxicity^b^		Grade 0–2	Grade 3–4	1.048(0.767–1.432)	0.768	1.034(0.636–1.679)	0.894	1.109(0.641–1.920)	0.711
	GG	69(18.9)	23(22.8)						
	GA	175(47.8)	45(44.6)						
	AA	118(32.2)	33(32.7)						
Hematological toxicity^b^		Grade 0–2	Grade 3–4	1.012(0.749–1.366)	0.94	0.965(0.611–1.523)	0.878	1.090(0.639–1.859)	0.751
	GG	69(19.5)	23(20.2)						
	GA	167(47.3)	53(46.5)						
	AA	113(32.0)	38(33.3)						

*Abbreviations* *n *number, *OR *Odds ratio, *CI *Confidence interval

^a^ with adjustments of age and sex;

^b^ with adjustments of age, sex, stage, histological type, smoking status, and chemotherapy regimens

**Fig. 1:**
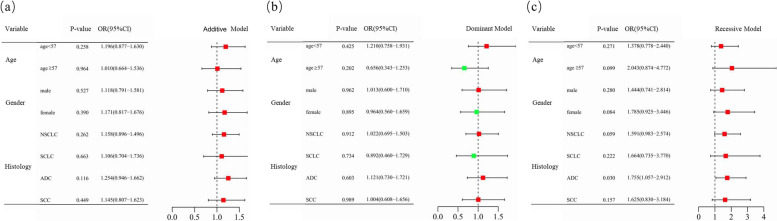
Stratification analyses of the association of *CCND1* rs9344 with lung cancer risk. **a**–**c** Additive (**a**), dominant (**b**), and recessive (**c**) models with adjustments of age and sex. Each box and horizontal line represent the odds ratio (OR) and 95% confidence interval (CI). NSCLC non-small cell lung carcinoma, ADC adenocarcinoma, SCC squamous cell carcinoma, SCLC small cell lung cancer

Subsequently, the stratified analyses were performed. As shown in Fig. 1 and Supplementary Table 3, *CCND1* rs9344 was significantly associated with adenocarcinoma (ADC) patients in the recessive model. The cancer susceptibility was higher in ADC patients with *CCND1* rs9344 AA genotypes than in those with GG and GA genotypes (adjusted OR = 1.755, 95%CI = 1.057–2.912, *P* = 0.030) (Fig. 1).

### Association of *CCND1* rs9344 and platinum-based chemotherapy response in lung cancer patients

Among the 498 cases of lung cancer, 467 of them had received more than two cycles of platinum-based chemotherapy. As shown in Table 1 and Supplementary Tables 1, 283 responders and 184 non-responders were included, respectively. The unconditional logistic regression analysis was conducted after adjusting for the age, sex, stage, histological type, smoking status and chemotherapy regimen. However, no significant correlation was identified between *CCND1* rs9344 polymorphism and platinum-based chemotherapy response (Table 2 and Supplementary Table 2) in the general overall pooled analysis.

**Fig. 2:**
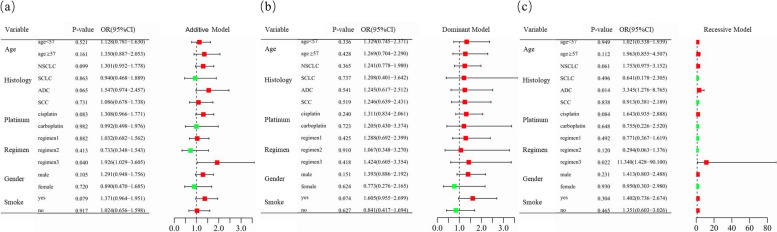
Stratification analyses of the association of *CCND1* rs9344 with platinum-based chemotherapy response. **a**–**c** Additive (**a**), dominant (**b**), and recessive (**c**) models with adjustments of age, sex, stage, histological type, smoking status, and chemotherapy regimens. Each box and horizontal line represent the odds ratio (OR) and 95% confidence interval (CI). NSCLC non-small cell lung carcinoma, ADC adenocarcinoma, SCC squamous cell carcinoma, SCLC small cell lung cancer. Regimen1, platinum + gemcitabine. Regimen2, platinum + etoposide. Regimen3, platinum + pemetrexed

However, *CCND1* rs9344 was found to be significantly correlated with the platinum-based chemotherapy response of patients who received platinum + pemetrexed therapy (additive model: adjusted OR = 1.926, 95%CI = 1.029–3.605, *P* = 0.040; recessive model: adjusted OR = 11.340, 95%CI = 1.428–90.100, *P* = 0.022). In addition, a significant correlation was also found between *CCND1* rs9344 and platinum-based chemotherapy response in the subgroup of ADC patients (recessive model: adjusted OR = 3.345, 95%CI = 1.276–8.765, *P* = 0.014) (Fig. 2 and Supplementary Table 3).

### Association of *CCND1* rs9344 with platinum‑based chemotherapy toxicity in lung cancer patients

**Fig. 3:**
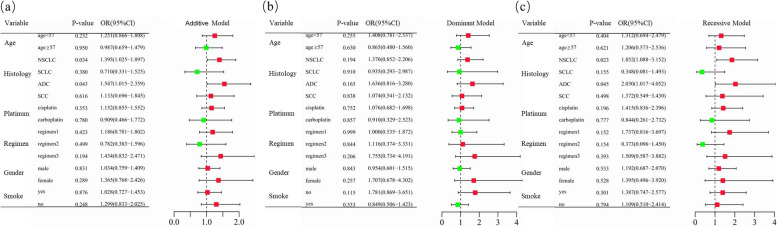
Stratification analyses of *CCND1* rs9344 and chemotherapy-induced overall toxicity in lung cancer patients. **a**–**c** Additive (**a**), dominant (**b**), and recessive (**c**) models with adjustments of age, sex, stage, histological type, smoking status, and chemotherapy regimens. Each box and horizontal line represent the odds ratio (OR) and 95% confidence interval (CI). NSCLC non-small cell lung carcinoma, ADC adenocarcinoma, SCC squamous cell carcinoma, SCLC small cell lung cancer. Regimen1, platinum + gemcitabine. Regimen2, platinum + etoposide. Regimen3, platinum + pemetrexed

**Fig. 4:**
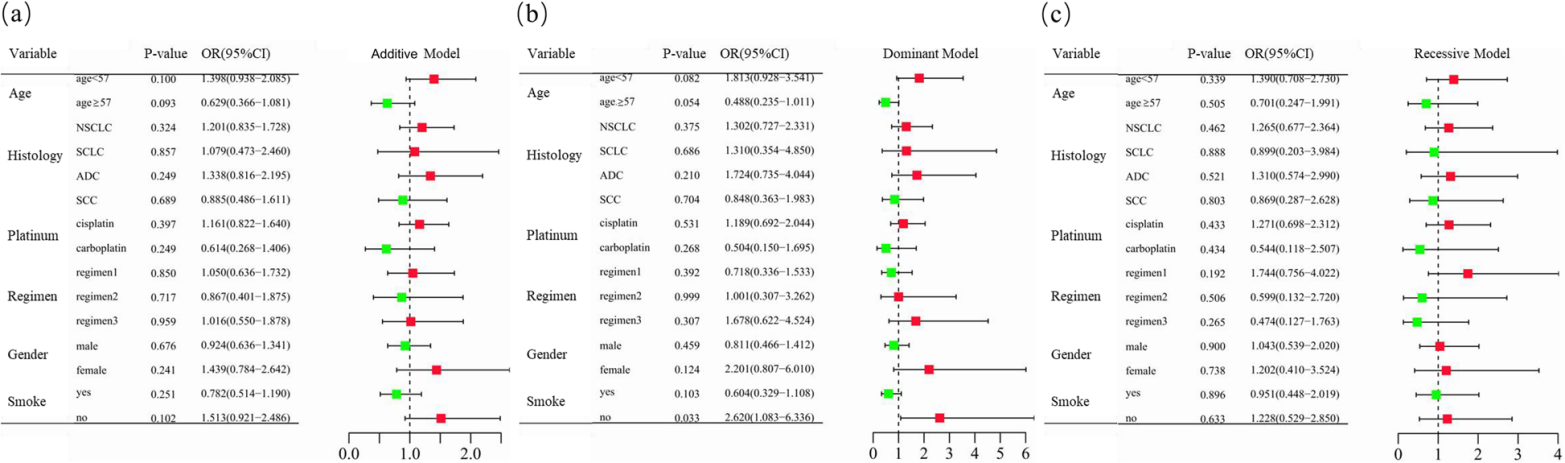
Stratification analyses of *CCND1* rs9344 and chemotherapy-induced gastrointestinal toxicity in lung cancer patients. **a**–**c** Additive (**a**), dominant (**b**), and recessive (**c**) models with adjustments of age, sex, stage, histological type, smoking status, and chemotherapy regimens. Each box and horizontal line represent the odds ratio (OR) and 95% confidence interval (CI). NSCLC non-small cell lung carcinoma, ADC adenocarcinoma, SCC squamous cell carcinoma, SCLC small cell lung cancer. Regimen1, platinum + gemcitabine. Regimen2, platinum + etoposide. Regimen3, platinum + pemetrexed

**Fig. 5:**
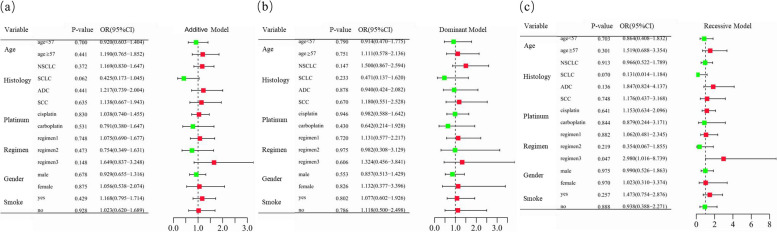
Stratification analyses of *CCND1* rs9344 and chemotherapy-induced hematological toxicity in lung cancer patients. **a**–**c** Additive (**a**), dominant (**b**), and recessive (**c**) models with adjustments of age, sex, stage, histological type, smoking status, and chemotherapy regimens. Each box and horizontal line represent the odds ratio (OR) and 95% confidence interval (CI). NSCLC non-small cell lung carcinoma, ADC adenocarcinoma, SCC squamous cell carcinoma, SCLC small cell lung cancer. Regimen1, platinum + gemcitabine. Regimen2, platinum + etoposide. Regimen3, platinum + pemetrexed

Of the 467 lung cancer patients who received more than two cycles of platinum-based chemotherapy, 181 had undergone at least one type of severe toxicity. Grade 3–4 gastrointestinal and hematologic toxicities occurred in 101 and 114 patients, respectively (Table 1 and Supplementary Table 1). Unconditional logistic regression analysis demonstrated no significant correlation between *CCND1* rs9344 and overall toxic reactions (Table 2 and Supplementary Table 2). However, *CCND1* rs9344 was significantly correlated with overall toxicity in NSCLC patients in both the additive model (adjusted OR = 1.395, 95%CI = 1.025–1.897, *P* = 0.034) and the recessive model (adjusted = 1.852, 95%CI = 1.088–3.152, *P* = 0.023). The same tendency was also observed in ADC patients, with a significantly increased incidence of overall toxicity in both the additive model (adjusted OR = 1.547, 95%CI = 1.015–2.359, *P* = 0.043) and the recessive model (adjusted OR = 2.030, 95%CI = 1.017–4.052, *P* = 0.045) (Fig. 3 and Supplementary Table 3). The two types of toxicities were then analyzed separately. *CCND1* rs9344 was significantly associated with an increased risk of gastrointestinal toxicity in non-smokers (recessive model: adjusted OR = 2.620, 95%CI = 1.083–6.336, *P* = 0.035) (Figs. 4 and 5 and Supplementary Table 3).

### Association of *CCND1* rs9344 with 5-year overall survival in lung cancer patients

**Fig. 6:**
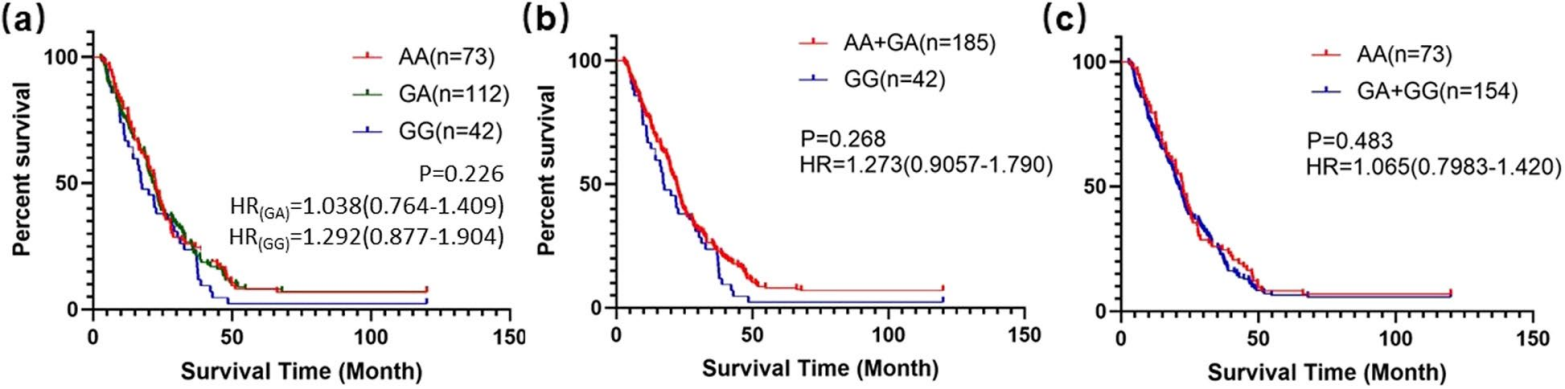
Genotype of *CCND1* rs9344 and its association with 5-year overall survival. **a** AA vs. GA vs. GG; **b** AA + GA vs. GG; **c** AA vs. GA + GG

Finally, we analyzed the correlation between *CCND1* rs9344 polymorphism and 5-year overall survival of lung cancer patients. Kaplan-Meier survival analyses were separately performed in three genetic models. Non-significant difference was observed in the 5-year overall survival rate between AA vs. GA vs. GG genotype patients (*P* = 0.226) (Fig. 6a). We also did not find any significant correlation in the dominant and recessive models (dominant model: HR = 2.268 (0.9057-1.790), *P* = 0.268; recessive model: HR = 1.065 (0.7983-1.420), *P* = 0.483). Results of multivariate Cox propotional hazards regression were exhibited in Supplementary Table 4.

### A meta-analysis elucidating the relationship between *CCND1* rs9344 and lung cancer susceptibility

**Fig. 7:**
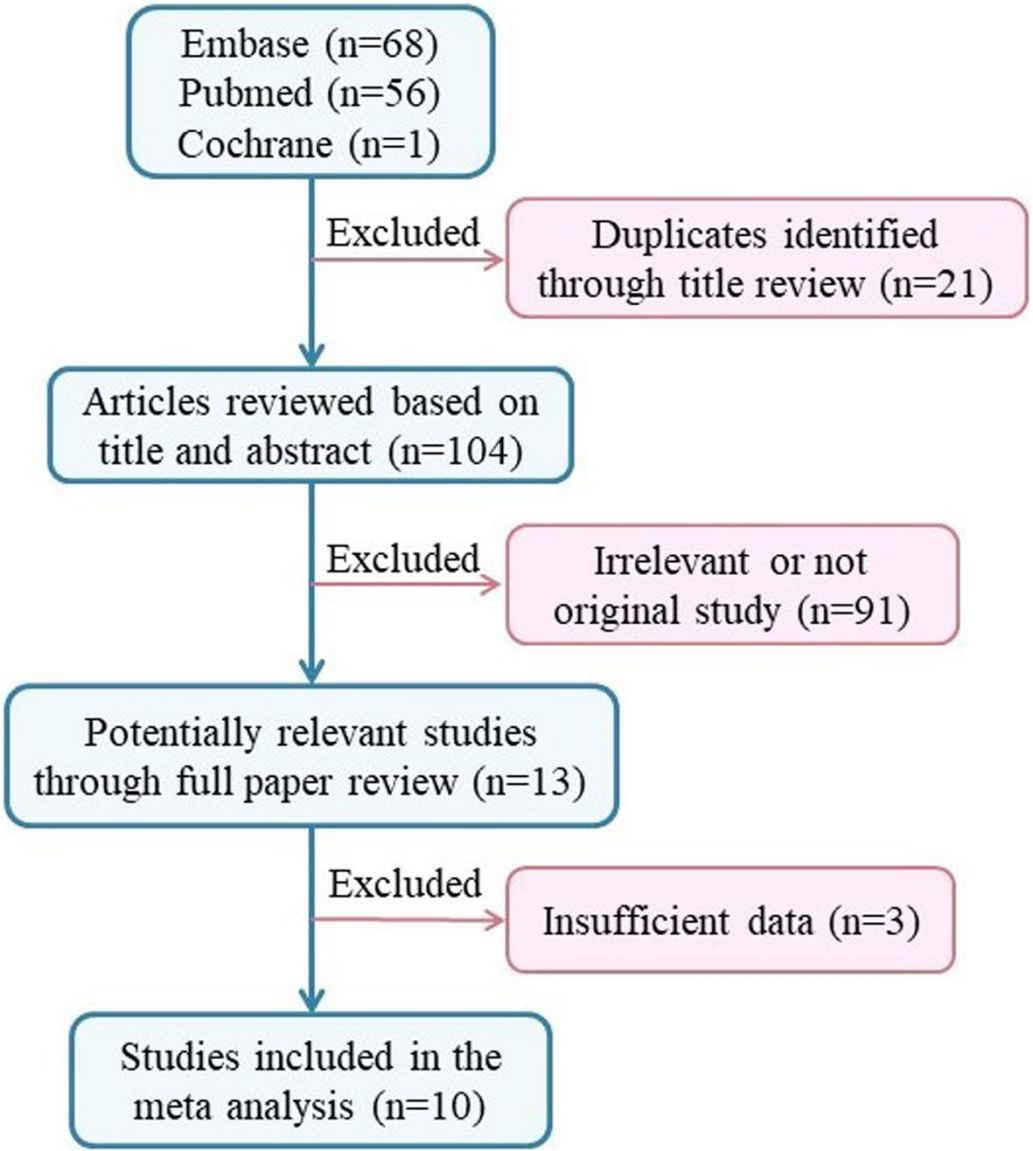
Flow chart of the study selection process

**Fig. 8:**
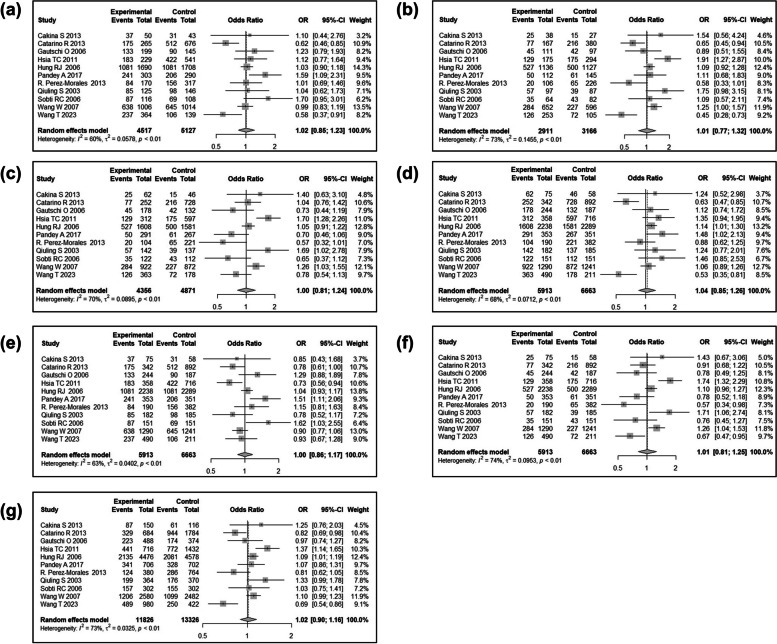
Meta-analyses of correlation between *CCND1* rs9344 and lung cancer risk under the random effects model. **a** Codominant1 (GA VS GG); **b** Codominant2 (AA VS GG); **c** Codominant3 (AA VS GA); **d** Dominant (AA + GA VS GG); **e** Overdominant (GA VS AA + GG); **f** Recessive (AA VS GA + GG); **g** Allelic (A VS G). The boxes and horizontal lines indicate the risk ratio (RR) and 95% confidence interval (CI), respectively

**Fig. 9:**
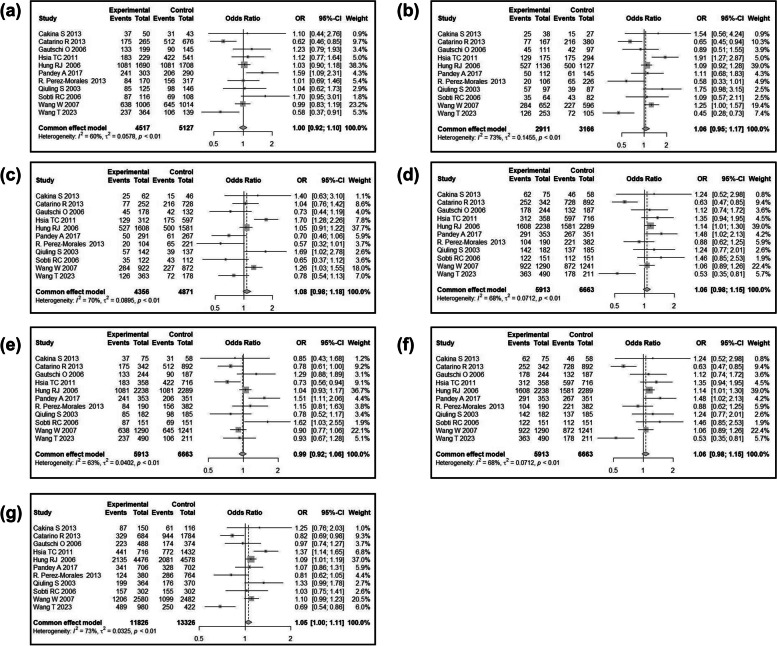
Meta-analyses of correlation between *CCND1* rs9344 and lung cancer risk under the fixed effects model. **a** Codominant1 (GA VS GG); **b** Codominant2 (AA VS GG); **c** Codominant3 (AA VS GA); **d** Dominant (AA + GA VS GG); **e** Overdominant (GA VS AA + GG); **f** Recessive (AA VS GA + GG); **g** Allelic (A VS G). The boxes and horizontal lines indicate the risk ratio (RR) and 95% confidence interval (CI), respectively

**Fig. 10:**
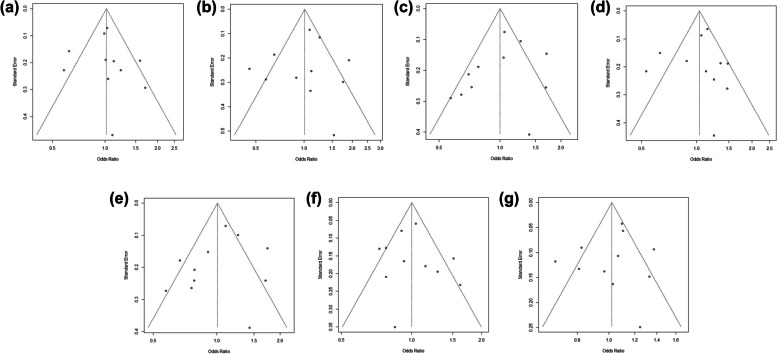
Funnel plot of *CCND1*rs9344 and lung cancer risk under the random effects model. **a** Codominant1 (GA VS GG); **b** Codominant2 (AA VS GG); **c** Codominant3 (AA VS GA); **d** Dominant (AA + GA VS GG); **e** Overdominant (GA VS AA + GG); **f** Recessive (AA VS GA + GG); **g** Allelic (A VS G)

**Fig. 11:**
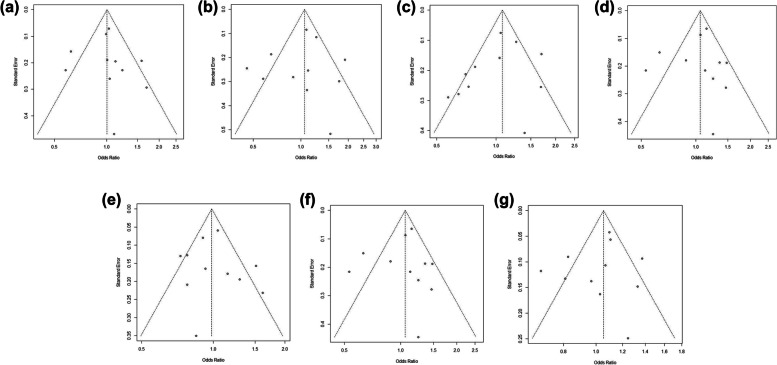
Funnel plot of *CCND1* rs9344 and lung cancer risk under the fixed effects model. **a** Codominant1 (GA VS GG); **b** Codominant2 (AA VS GG); (**c**) Codominant3 (AA VS GA); **d** Dominant (AA + GA VS GG); **e** Overdominant (GA VS AA + GG); **f** Recessive (AA VS GA + GG); **g** Allelic (A VS G)

**Fig. 12:**
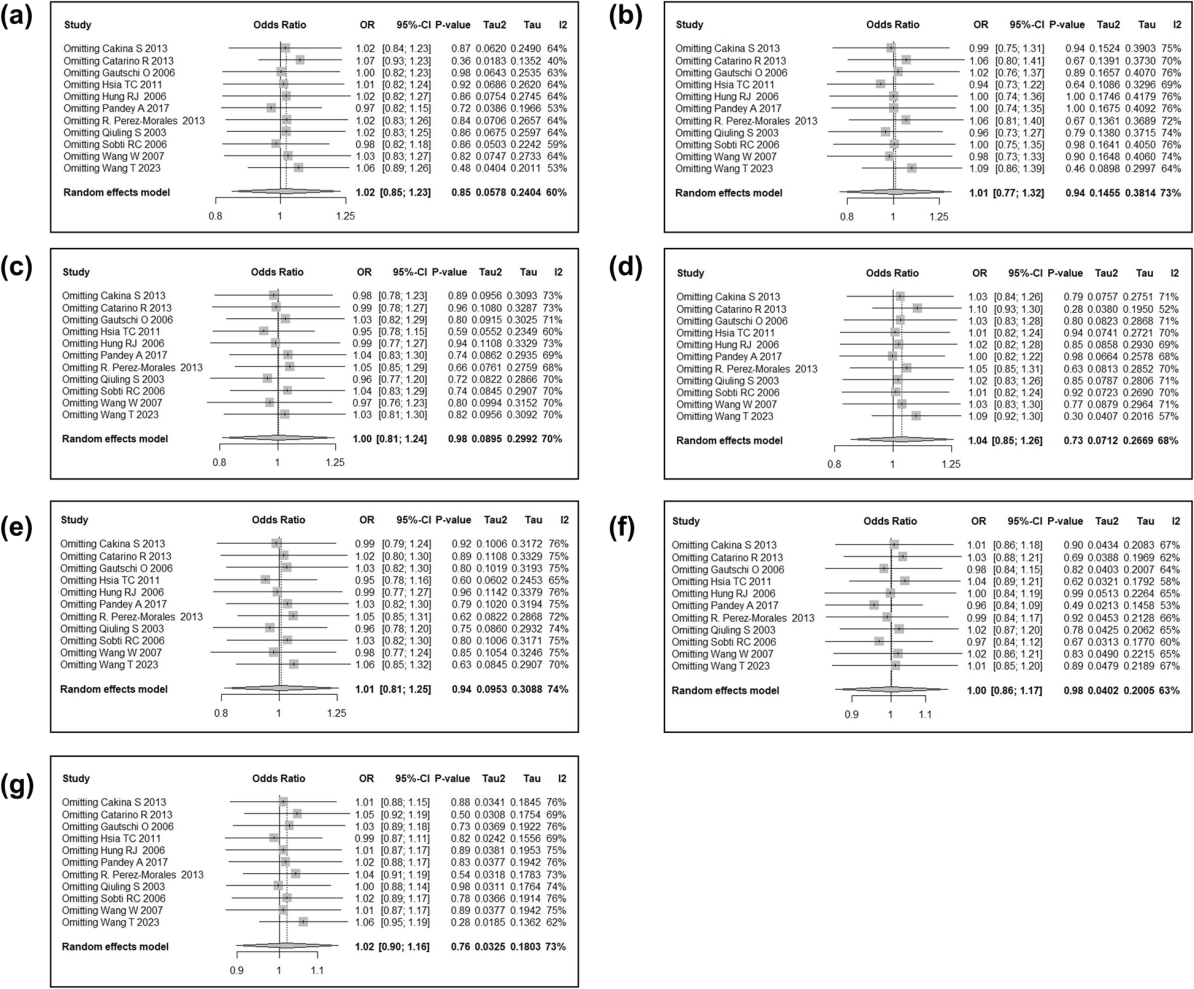
Funnel plot of sensitivity analyses of meta-analysis. The sensitivity analyses were performed by omitting one included study at a time. The boxes and horizontal lines indicate the risk ratio (RR) and 95% confidence interval (CI), respectively

We then conducted a meta-analysis to assess the association between *CCND1* rs9344 and lung cancer susceptibility. Following the process exhibited in Fig. 7, a total of 104 relevant studies were retrieved according to the search formula, and 10 of them were finally included according to inclusion criteria. Table 3 summarized the characteristics of the selected studies evaluating the association of *CCND1* rs9344 with lung cancer susceptibility. A total of 5432 cases and 6452 control samples were included. As seen in Table 4, the overall OR with 95%CI did not indicate significant differences in the lung cancer risk in random effects (Fig. 8) and fixed effect models (Fig. 9). The funnel plots were used to check the publication bias, which indicated that there was no significant publication bias (Figs. 10 and 11). Both the Begg’s *P*-value and the Egger’s *P*-value were not significant (Table 4). Sensitivity analyses were performed to check the robustness of the meta-analysis results by neglecting one included study at a time. As shown in Fig. 12, no single study was found to significantly influence the summary results.

**Table 3:** Characteristics of the included studies on CCND1 rs9344 polymorphisms and cancer susceptiblity

First Author	Year	Country	Ethnicity	Cancer type	Source of control	Genotyping method	NOS score	Number of cases	Number of control	Genotype of cases			Genotype of controls			Ref
Cakina S	2013	Turkey	Caucasian	Lung cancer	Unknown	PCR-RFLP	5	75	58	13	37	25	12	31	15	[43]
Catarino R	2013	Portugal	Caucasian	NSCLC	PB	PCR-RFLP	6	342	892	90	175	77	164	512	216	[42]
Gautschi O	2006	Switzerland	Caucasian	NSCLC	PB	PCR-RFLP	6	244	187	66	133	45	55	90	42	[41]
Hsia TC	2011	China	Asian	Lung cancer	HB	PCR-RFLP	7	358	716	46	183	129	119	422	175	[40]
Hung RJ	2006	Europe	Caucasian	Lung cancer	HB	TaqMan	7	2238	2289	609	1081	527	627	1081	500	[39]
Pandey A	2017	India	Caucasian	Lung cancer	HB	PCR-RFLP	5	353	351	62	241	50	84	206	61	[38]
R. Pe´rez-Morales	2013	Mexico	Caucasian	Lung cancer	Unknown	PCR-RFLP	5	190	382	86	84	20	161	156	65	[44]
Qiuling S	2003	China	Asian	Lung cancer	PB	PCR-SSCP	7	182	185	40	85	57	48	98	39	[45]
Sobti RC	2006	India	Caucasian	Lung cancer	HB	PCR-RFLP	4	151	151	29	87	35	39	69	43	[46]
Wang W	2007	American	Caucasian	Lung cancer	HB	TaqMan	7	1290	1241	368	638	284	369	645	227	[47]

*Abbreviations* *HB *Hosipital-based, *NSCLC *Non-small cell lung cancer, *PB *Population-based, *Ref *Reference

**Table 4:** *CCND1* rs9344 and lung cancer risk under the random- and fixed- effects model

Genetic model	Random-effects model					Fixed-effects model					Publication bias (P)	
	Test of association			Test of heterogeneity		Test of association			Test of heterogeneity			
	OR	95%CI	* P *	* P *	I^2^(%)	OR	95%CI	* P *	* P *	I^2^(%)	Egger’s test	Begg’s test
*GA* vs. *GG*	1.02	0.85–1.23	0.847	0.0052	60.10%	1.00	0.9207–1.0957	0.9215	0.01%	60.10%	0.7850	0.3502
*AA vs. GG*	1.01	0.77–1.32	0.943	< 0.0001	73.10%	1.06	0.9522–1.1727	0.2989	< 0.0001	73.10%	0.6488	1.0000
*AA vs. GA*	1.00	0.81–1.24	0.983	0.0002	69.90%	1.08	0.9833–1.1788	0.1106	0.0002	69.90%	0.3409	0.2758
*AA + GA vs. GG*	1.04	0.85–1.26	0.726	0.0005	68.20%	1.06	0.9755–1.1478	0.1735	0.0005	68.20%	0.7651	0.8763
*GA vs. AA + GG*	1.01	0.86–1.17	0.976	0.0025	63.20%	0.99	0.9175–1.0590	0.6938	0.0025	63.20%	0.7450	0.5334
*AA vs. GA + GG*	1.00	0.81–1.25	0.942	< 0.0001	73.80%	1.09	0.9977–1.1844	0.0564	< 0.0001	73.80%	0.3367	0.5334
allelic *A* vs. *G*	1.02	0.90–1.16	0.757	< 0.0001	73.30%	1.05	1.0005–1.1074	0.0478	< 0.0001	73.30%	0.5028	0.8763

*Abbreviations* *n *number, *OR *Odds ratio, *CI *Confidence interval

## Discussion

Lung cancer remains one of the leading disease burdens. While the last two decades have witnessed the emergence of novel therapeutic approaches such as targeted therapy and immunotherapy, platinum-based chemotherapy remains the most widely employed treatment for lung cancer patients. However, only a subset of patients could benefit from platinum-based chemotherapy, while the others, who prove insensitive to platinum drugs, endure the burdens of toxic side effects without any associated improvement in survival outcomes. Deeper insight into the pathogenesis, discovery of predictive biomarkers and optimization in therapeutic methods may efficiently improve the treatment outcome [48–50]. Based on this, one of the issues that urgently need to be addressed now discovering reliable biomarkers to identify individuals with a higher sensitivity to platinum-based chemotherapy. This expansion may provide promising possibilities for lung cancer diagnosis, treatment and prevention.

Unbalanced cycle regulation is one of the hallmarks of carcinogenesis. Cyclin D1 plays a crucial role in the transition from the G1 to the S phase of the cell cycle, thus being widely recognized as a pivotal element during the malignant transformation process [51]. The rs9344 (A870G), located in exon 4 of *CCND1* gene, is a frequent gene polymorphism that regulates alternative splicing and enables the expression of the transcribed Cyclin D1b. The prediction value of *CCND1* rs9344 in the prognosis of lung cancer patients has been investigated in several previous studies. However, few of them concentrated on platinum-based chemotherapy response. Hsia, et al. reported that among the lung cancer patients and cancer-free healthy controls, genotype distribution (*P* = 0.0003) and allelic frequency (*P* = 0.0007) of *CCND1* rs9344 were significantly different. Individuals who carried the AG and GG genotypes had a 0.59- and 0.52-fold risk of lung cancer compared to the AA genotype, respectively (95% CI, 0.44–0.78 and 0.35–0.79) [40]. Sobti et al. also indicated that the AG genotype was correlated with a higher risk of lung cancer (OR = 1.7, 95% CI = 0.92–3.14) [46]. Gautschi, et al. found that *CCND1* GG genotype was significantly correlated with platinum-based chemotherapy response (*P* = 0.04), while no significant difference was identified in patients’ prognosis among different genotypes [41]. However, Cakina, et al. indicated that no correlation was found in *CCND1* A870G polymorphism between lung cancer patients and controls [43].

This study conducted a hospital-based case-control investigation focusing on lung cancer, and systematically investigated the association between *CCND1* rs9344 and lung cancer susceptibility, platinum-based chemotherapy sensitivity, toxicity, and overall survival. While no significant differences were observed in the general population, the predictive potential of CCND1 rs9344 was established within specific patient subgroups. For cancer susceptibility, patients with the AA genotype exhibited a significantly higher risk than those with the GG + GA genotype (recessive model, adjusted OR = 1.755, 95%CI = 1.057–2.912, *P* = 0.030). In the context of platinum-based chemotherapy, *CCND1* rs9344 showed significant correlations with therapy response in patients receiving the PP regimen (additive model: adjusted OR = 1.926, 95%CI = 1.029–3.605, *P* = 0.040; recessive model: adjusted OR = 11.340, 95%CI = 1.428–90.100, *P* = 0.022). This significant association was also observed among ADC patients (recessive model: adjusted OR = 3.345, 95%CI = 1.276–8.765, *P* = 0.014). Furthermore, an increased risk of overall toxicity was found in both NSCLC (additive model: adjusted OR = 1.395, 95%CI = 1.025–1.897, *P* = 0.034; recessive model: adjusted OR = 1.852, 95%CI = 1.088–3.152, *P* = 0.023) and ADC patients (additive model: adjusted OR = 1.547, 95%CI = 1.015–2.359, *P* = 0.043; recessive model: adjusted OR = 2.030, 95%CI = 1.017–4.052, *P* = 0.045). Notably, in non-smokers, *CCND1* rs9344 was significantly associated with a higher risk of gastrointestinal toxicity (adjusted OR = 2.620, 95%CI = 1.083–6.336, *P* = 0.035).

In addition to the case-control study, a comprehensive meta-analysis for previous research on *CCND1* rs9344 and lung cancer susceptibility was conducted. In line with our findings, no significant correlation was observed on a overall scale. This may arise from various factors such as variations in sample selection and distribution, disparities in research quality, substantial heterogeneity in environmental factors, or gene-environment interactions. The results of our study and meta-analysis consistently suggest that the predictive role of *CCND1* rs9344 in therapeutic efficacy and prognosis of lung cancer patients may not be effective for all individuals, but rather requires more precise subgroup analysis. Besides, the lack of statistical significance at the overall level may also be caused by various factors in different studies, including differences in sample selection and distribution, variations in study quality, substantial heterogeneity of environmental factors, or gene-environment interactions. The predictive value of *CCND1* rs9344 remains to be further validated in large samples through stratified analysis.

## Conclusion

To summarize, this study demonstrated that *CCND1* rs9344 may be considered a candidate biomarker for cancer susceptibility and therapeutic outcome in certain patient subgroups in Chinese population. Further stratified studies with larger sample sizes are needed to confirm the results.

### Supplementary Information

**Supplementary Material 1.**

## Data Availability

The data presented in this study are available on request from the corresponding author.

## Abbreviations

ADC: Adenocarcinoma
CR: Complete response
PD: Progressive disease
PR: Partial response
SD: Stable disease
SNPs: Single nucleotide polymorphisms
